# Some of the Dangers of an Impure Meat and Milk Supply

**Published:** 1899-11

**Authors:** W. H. Dalrymple


### SOME OF THE DANGERS OF AN IMPURE MEAT AND MILK SUPPLY.*

BY W. H. DALRYMPLE, M.R.C.V.S.

The success of all great reforms is largely dependent upon the education of public opinion. Ignorance is the mighty incubus which retards the advancement of all progressive movements. Science has had to struggle on through the ages against the enormous odds of incredulity, indifference, and superstition; but truth will prevail; and scientific investigation, which is but the searching after truth, although hampered on all sides by those influences which are born of ignorance, superstition and doubt, is slowly but surely revealing the true nature of things, and must eventually and effectually succeed, but the rate of progress, in the accomplishment of the desired end, will greatly depend upon the acceptance of the revealed truths by the people, which can only be attained by the education of public opinion.

There is no education more valuable in its possession, nor more philanthropic in its aim, than that which has for its object the amelioration of the conditions of our fellow men. Life without health is an emasculated existence, shorn of everything that goes to make up the pleasure of living; and the individual, the association, or the community of whom it can be said that he, or they, inaugurated, or stood up for reforms that resulted in the preservation of health, or the prolongation of even one human life, has accomplished a work the value of which can hardly be estimated.

What are the purposes and objects of this association? They may be summed up in the following sentence: The conservation of public health and the prolongation of human life; and its hope of success lies mainly in being able to place before the people scientific facts along the various branches of Sanitary Science, in a way they can be received and intelligently acted upon; so that by concert of action, much needed reforms may be brought about, disease lessened, death made more remote, and health and happiness vouchsafed a more extended reign.

*Read before the Louisiana State Sanitary Association at Baton Rouge, June, 1899.

It has been stated by some authority that disease is more dependent upon food and water than upon all other influences combined. This seems a very astounding assertion, when we consider that life itself is also dependent upon the same. It must be understood, however, that there is a very great deal of difference between wholesome food and water and that which is capable of producing disease; and it is a fact of the utmost importance, and one which ought to be indelibly impressed upon the mind of every one, that although both, in a pure and wholesome condition, are necessary to life and health, they also form suitable media for the cultivation and development of various pernicious agencies, in the form of pathogenic bacteria, and other parasitic forms, which render them especially dangerous as food articles for human consumption. It is along this line of thought that I desire to ask your attention for a short time, and I would like to lay special stress upon the desirability of securing for city populations a meat and milk supply that is as pure and wholesome as it is possible to make it.

Let us for a moment consider some of the agencies that render those articles of human food inimical to the health of the consumer. I will only mention a few, but quite sufficient to emphasize their importance as a source of danger to public health.

I shall be forced to refer to some facts which may be somewhat repugnant to our finer feelings, but as they are stern realities, and there is no getting beyond them, it is only right that we should be made familiar with them. It may be all very well to be possessed with the comfortable feeling, that, "where ignorance is bliss, tis folly to be wise." This may do so long as there is nothing of any particular value at stake; but when human life and health are in the balance, we cannot know too much of the many untoward influences which militate against their fullest vigor; and it is only by laying bare plain facts regarding the predisposing and exciting causes of diseases, that we can expect public opinion to be educated along these lines, or hope for their ultimate eradication through combined and intelligent effort.

Although the great majority of us have been in the habit, all our lives, of eating with a relish our beefsteak, our pork-chop, or, perhaps, some daintily spiced sausage-meat, yet the minority only may have any conception that our meat animals are capable of being the bearers of a number of diseases, some of which are of a fatal character to the human family.

Let us consider one or two of the more important, and, as I have previously suggested, throw aside sentiment for the time being, and view them from a matter-of-fact standpoint—the standpoint of public health.

Swine, which furnish a large proportion of our meat supply, besides being subject to numerous other diseases, are capable of harboring, and, in fact, do harbor several parasites which are transmitted to human beings through consumption of their flesh, in an imperfectly cooked condition, which often produce serious consequences.

The muscles or flesh of this valuable food animal furnishes the habitat for an important parasite, in a partially developed state, which, when partaken of with the flesh which has not undergone sufficient cooking to destroy it, comes to maturity in the human alimentary canal, and there produces what is technically known as "tæniasis," but which, in plain, ordinary language, means nothing more nor less than tape-worm disease.

The hog is responsible, also, for affording asylum to another parasite, which, although about the smallest of the nematode or round worms, is mighty enough in its effects to cause embargoes to be at times placed by foreign governments upon our export hog products.

I refer here to the "trichina spiralis," which infests the muscular tissues of the hog, and when the flesh is eaten, without undergoing sufficient heat, the consumer becomes affected with that often fatal disease known as "trichinosis" or flesh-worm disease.

Our beef animals, too, are responsible for the transmission of tæniasis to the human consumer in a similar way to that of the hog. Such beef is popularly known as "measly beef," and in the case of swine, "measly pork."

It may be a revelation to some to know that consumption is as prevalent, relatively, in cattle as it is amongst the human family. The germ or bacterium which produces the disease in the one case is similar to that which causes it in the other. In fact, consumption is considered to be intercommunicable.

Many of us view this subject lightly, not having devoted to it much thought; nevertheless the question of how to prevent consumption is one which, at the present time, is occupying the serious attention of medical and veterinary sanitary scientists all over this country, and, in fact, throughout the entire civilized world. Only within the last month there was held in the city of London, England, a statutory general meeting of the National Association for the prevention of consumption and other forms of tuberculosis, presided over by the Earl of Derby, and at which a large number of the most eminent sanitarians of Great Britain were in attendance. The report of the organizing committee, which was read by the secretary, stated that they sought to enlist sympathy and cooperation by (1) the education of public opinion and the stimulation of individual initiative; (2) the influencing of Parliament, county councils, boards of guardians, chambers of agriculture, and other public authorities on matters relating to the prevention of tuberculosis; (3) the establishment throughout the kingdom of local branches of the association to be affiliated with the central office.

In the education of public opinion they suggested the circulation of pamphlets and leaflets setting forth in plain language the results of scientific investigation, public lectures by men approved by the council, addresses at congresses, and other public gatherings, cooperation with other societies having for their object the promotion of public health, and the establishment of open air sanatoria for tuberculous patients, etc. I have referred to this report to show the importance that is attached in other countries to the prevention and eradication of this "great white plague." I was much struck, also, with the similarity in the objects and the methods sought to enlist sympathy and cooperation in that powerful British association and in our own, as can be seen by referring to the charter of this organization.

Going back to our beef animals, we would say, that in the consumption of tuberculous flesh there is danger more or less to human life, and we do not believe that any one would relish a steak from a tuberculosis animal if they were aware of the fact. Meat, although

2

not actually diseased itself or from an animal that may have suffered or, perhaps, died from some communicable disease, may yet be unfit for human consumption on account of emaciation depriving it of proper nutritive qualities, and there are certain periods in an animal's life, although it may not be suffering from actual disease, in which its flesh is rendered repulsive as an article of human food.

Such, then, are a few of the conditions of our pork and beef animals through which consumers of their flesh may suffer, either from being actually diseased, or from want of the proper nourishing properties of the meat. But besides the meat the purity and wholesomeness of the milk supply of our city populations is no less important a necessity.

I will not trouble you with details relative to the various causes which render milk either nocuous or innutritious, but will refer to but one condition, on account of which this "great uncooked food" becomes extremely dangerous to life, and especially that of children. I refer to tubercular milk, or milk from consumptive cows.

Extensive statistics go to show that increase in the number of cases of consumption of the bowels of children (tabes mesenterica) is coincident with the time at which they are changed from their mother's milk to that of the cow. It is not the case that the bacillus of tuberculosis is found in the milk of every consumptive cow; but when the milk-vessel, the udder or bag, is affected with the disease, the milk from that animal is extremely dangerous, and where a herd of dairy cattle is kept, and consumption exists amongst them, it is possible, if not probable, that one of them may have tuberculosis of the udder, and by mixing the milk of this one cow with that of the others, the whole of the supply may thereby be contaminated with tubercle bacilli.

In order that I may not occupy too much time in discussing this subject, although it is one of immense moment, I will take the liberty of quoting a few statistics recently given in a valuable paper by an English medical officer of health, and which I trust may serve the purpose of emphasizing the fact that there is no lack of evidence of the causation of human tuberculosis by cow's milk. Among other things, Dr. Harold Scurfield (the gentleman referred to), medical officer for Sunderland, a large town in the north of

England, says: There are two reasons why the eradication of bovine tuberculosis is desirable. First, in the interest of agricultural communities, and, second, because its eradication necessarily entails a large reduction in the prevalence of one of the most fatal diseases of childhood, namely, tuberculosis or consumption of the bowels.

In enumerating some of the evidence as to the causation of human tuberculosis by cow's milk, Dr. Scurfield cites the following few striking features in the chain: (1) Tuberculosis in cattle and in man is the same disease, and is caused by the entrance into and growth in the body of the tubercle bacillus. The entrance of the bacillus into the body is so rarely effected before birth, that for all practical purposes it is true to say that every case of tuberculosis is due to infection. And only a day or two ago, at the tuberculosis congress held at Berlin, Prof. Rudolph Virchow, in an address before that body, rejected the theory of hereditary tuberculosis. This doctrine he declared, was contradicted by all his pathological researches. He said he had never found tuberculosis in unborn or new-born infants, though it might be contracted during the first day's existence.

(a) Cows suffering from tuberculosis of the udder give milk containing tubercle bacilli.

(3) Tuberculosis of the udder is not rare in cows, and milk, as ordinarily sold, often contains tubercle bacilli. Thus Bang, of Copenhagen says: In any large herd in which tuberculosis is rife, one will be certain to find one cow, the milk of which contains tubercle bacilli, and which has tuberculosis of the udder.

Out of 144 samples of milk coming from Liverpool cow stables, 4, or 2.8 per cent. were found to contain the germ of consumption, but of 24 samples of "country milk" taken at Liverpool railroad depots, 7, or 29 per cent. were found to contain the organism.

In some later experiments in Liverpool, 12 out of 228 samples of city milk (5.2 per cent.) were found to contain the germ, and 9 out of 67 samples of country milk, or 13.4 per cent.

Similar results were obtained at Cambridge. In Manchester 17 out of 93 samples of country milk were found to contain the bacillus. By permission of the farmers, the city veterinary officer visited 16 farms from which the milk came, and on 14 of these farms he found at least one cow with tuberculosis of the udder.

(4) Animals, such as calves, hogs, rabbits, guinea-pigs, cats and dogs, fed on milk from cows suffering from tuberculosis of the udder, develop tuberculosis of the bowels.

(5) Human beings contract consumption of the bowels just at the period of life when cow's milk forms the principal item in their diet.

(6) The sanitary improvements of the last forty years have reduced the death-rate from tuberculosis of the lungs by 45 per cent., or nearly one-half.

During the same period the death-rate from consumption of the bowels among infants under one year old has increased by 27 per cent., and this increase has been coincident with an increase in the proportion of infants raised on the bottle instead on their natural nutriment. Tuberculosis of the breast is rare in the human mother, but tuberculosis of the udder is comparatively common in the cow. We have then to contemplate the suggestive fact that the transfer of the infant from the rarely tuberculosis breast of the mother to the frequently tubercular udder of the cow has been coincident with an increase in the prevalence of tuberculosis of the bowels, and that this increase has taken place during a period when the prevalence of other forms of tuberculosis in the human being have been rapidly decreasing.

Such facts as these, and we could find plenty more evidence to back them up, here in our own country, should impress themselves not only upon the minds of those who have charge of the health of our citizens, but on the citizens themselves, for, as I have previously stated, it is only by concentrated effort that the necessity for such important reforms as, indicated by these statements, can be sufficiently appreciated or their achievement made an established fact.

How then are we to bring about the much-needed reforms and secure for our citizens a pure and wholesome meat and milk supply? The matter has received a great amount of attention and study by the leading sanitarians, both of this country and Europe, with the result that in the most progressive centers of population of both continents, it has been found necessary, in order to obtain the greatest amount of success, to consolidate slaughter-houses into one or more—according to requirements—central abattoirs, placed under municipal control; the dairies also, supplying city populations, under similar control, with inspection of both placed in the hands of an individual or individuals, as the requirements may demand, who has been specially trained for such work.

What would be the effect of such a system, it might be asked? We have but to look at the records of other cities that have adopted it to find that it has proved itself a boon to the consumer and a benefit to the vendor. Those who are engaged in providing meat and milk for human consumption, and especially in our centers of population, are, generally speaking, citizens of the highest honor and integrity, and would not, if they were aware of it, place on the market, for human food, either meat or milk that they did not consider perfectly sound and nutritious; but for the lack of the necessary pathological knowledge, the purveyor of these articles is unable to identify conditions which would be at once observed by the trained eye of the expert, and known by him to be injurious. Besides the former has, as a rule, little or no appreciation of the great importance, from a public health standpoint, of rigid sanitary conditions and surroundings, and even if he did, he has not the facilities for such in the ordinary butcher-pen or the common cow stable or dairy. Nor does he realize the danger of the contamination of his products resulting from insanitation; whereas the expert, who has been trained in hygiene and sanitation, is at once familiar with the various agencies and pathologic conditions that are likely to bring about disease. How much better and more satisfactory it must be for the butcher, who is an honorable man, and not afraid to expose his methods of work or his wares, to have his work done in an abattoir fitted up with all the most modern improvements and appliances to insure speed and cleanliness, than to labor in a place, the whereabouts of which can often be easily discovered without the aid of a guide, or the milk vendor who is cognizant of the fact, by actual test, that his cows are free from tuberculosis, and that his milk has been handled under the most approved sanitary precautions, and reaches the customer positively pure and wholesome. And how much more comfortable must be the feelings of the consumer who sits down with his family to his roast of beef, or pork, or other meat product, or his pitcher of milk, when he knows that they have been produced by animals that are not only free from disease, but in a fit condition to slaughter for food, or to yield milk for censumption, and that they have been handled with the greatest sanitary care; have passed before the scrutinizing eye of a competent inspector, and come to his table stamped with the mark of purity on them.

I have not the time on this occasion to go into details with regard to the construction and working of a public abattoir, or a meat and milk inspection under municipal control. My object at present is, chiefly to set before you a few of the too-frequently overlooked dangers to public health, which frequently lurk in the flesh of some of the more important of our animal meats, and in the milk of our dairy herds, that you may the more fully appreciate the necessity for the inauguration of a system, where it does not already exist, that will insure to the consumer these valuable food materials in an absolutely pure and wholesome condition.

It is only a matter of time when every city and town of any size in the country will have its public abattoir, with its meat and milk inspection under municipal control. Public opinion is fast being educated along this particular line, and when it becomes more generally known that some important diseases can be transmitted to human beings through the consumption of unsound meat and milk, under the old, and, in many cases, the present insanitary system, and that such disease can be positively prevented by adopting the new and more sanitary system, the latter is going to be demanded.

Outside of the city of New Orleans, which has had a meat and milk inspection for several years, the city of Shreveport is taking the lead. She is now engaged in, or is about to commence, the erection of a modern building to be under the control of the city Board of Health, where all animals that are intended to supply meat to her citizens will have to be slaughtered and their flesh inspected before being allowed to be placed on the market as an article of human food. This will insure to them a meat supply that is not only free from disease, but also a food that is nutritious and wholesome.

Why, I might ask, should our capital city be behind the city of Shreveport? Our citizens are quite as progressive, I hope, and I feel sure they are just as epicurean in their tastes. This subject should in reality enlist the serious consideration of the citizens of every community in the State, but it appears to me to be extremely opportune that we should devote to it special thought at the present time. We have in contemplation extensive improvements for our city along sanitary lines. We have just had the indorsement of our citizens to the imposition of a tax for the purpose of securing sufficient money to carry out these improvements. Why not add to the list a public abattoir, and the other necessaries required to insure a meat and milk supply that would not only be pure and wholesome, but for quality, also, would be second to none in the country? It would be a lasting monument to the intelligence and progressiveness of the citizens of our town, and with the result that no one would suffer financially, but all would benefit from the all-important standpoint of public health.

